# Human coronavirus NL63: a clinically important virus?

**DOI:** 10.2217/fmb.10.166

**Published:** 2011-03-02

**Authors:** Burtram C Fielding

**Affiliations:** 1 Molecular Virology Research Laboratory, Medical Microbiology Cluster, Department of Medical Biosciences, Faculty of Natural Sciences, University of the Western Cape, Private Bag X17, Modderdam Road, Bellville, Western Cape, 7535, South Africa. Tel.: +27 219 593 620; Fax: +27 219 593 125; bfielding@uwc.ac.za

**Keywords:** childhood viral infections, clinical features, HCoV-NL63, human coronavirus, lower respiratory tract infection, respiratory viruses, upper respiratory tract infection

## Abstract

Respiratory tract infection is a leading cause of morbidity and mortality worldwide, especially among young children. Human coronaviruses (HCoVs) have only recently been shown to cause both lower and upper respiratory tract infections. To date, five coronaviruses (HCoV-229E, HCoV-OC43, SARS-CoV, HCoV-NL63 and HCoV HKU-1) that infect humans have been identified, four of which (HCoV-229E, HCoV-OC43, HCoV-NL63 and HCoV-HKU-1) circulate continuously in the human population. Human coronavirus NL63 (HCoV-NL63) was first isolated from the aspirate from a 7-month-old baby in early 2004. Infection with HCoV-NL63 has since been shown to be a common worldwide occurrence and has been associated with many clinical symptoms and diagnoses, including severe lower respiratory tract infection, croup and bronchiolitis. HCoV-NL63 causes disease in children, the elderly and the immunocompromised, and has been detected in 1.0–9.3% of respiratory tract infections in children. In this article, the current knowledge of human coronavirus HCoV-NL63, with special reference to the clinical features, prevalence and seasonal incidence, and coinfection with other respiratory viruses, will be discussed.

Acute respiratory tract infections (ARTIs) are among the most common causes of disease in humans [1]. The majority of ARTIs are caused by viruses with rhinovirus, respiratory syncytial virus, influenza virus, enterovirus, human metapneumovirus and parainfluenza virus considered the major pathogens [1–3]. Those most at risk of severe complications from these viral infections include young children, the elderly or persons with compromised cardiac, pulmonary or immune systems [2,4]. The high burden of disease caused by respiratory viruses in young children has led to the development of diagnostic tests and vaccines for the treatment of these infections [5].

Until recently, it was commonly accepted that the known human coronaviruses (HCoVs), with the exception of severe acute respiratory syndrome Cov (SARS-CoV), mainly cause mild upper respiratory tract infections (URTIs) [6]. For this reason, the circulation of HCoVs was not monitored and no attempt to develop vaccines or drugs against these viruses was made [7]. CoVs are ssRNA viruses that infect humans and animals. In animals, CoVs cause a wide spectrum of diseases, including respiratory, enteric, hepatic and neurological diseases, with symptoms ranging from mild to severe [8,9]. HCoVs causing URTIs were first isolated from patients in the 1960s [8], with HCoV-229E and HCoV-OC43 the best characterized. Then, following the outbreak of SARS in China in 2003, three additional human coronaviruses were identified – SARS-CoV [10–12], HCoV-NL63 [13,14] and HCoV-HKU1 [15]. Of the five known HCoVs, four (HCoV-229E, HCoV-OC43, HCoV-NL63 and HCoV-HKU1) are circulating continuously in the human population.

This article aims to summarize the current knowledge of HCoV-NL63 with reference to the clinical features, prevalence and seasonal incidence, and coinfection with other respiratory viruses. Finally, perspectives for future developments in the field are discussed.

## Prevalence & seasonal incidence

HCoV-NL63 was first isolated from a 7-month-old baby with bronchiolitis in early 2004 [13]; at approximately the same time, essentially the same virus (named HCoV-NL) was isolated from an 8-month-old boy suffering from pneumonia [16]. The virus has since been detected in 1.0–9.3% of respiratory tract samples collected in different countries indicating a global distribution (Table 1) [5,17–33].

In a study testing the serum samples of 139 children, HCoV-NL63 seroconversion occurred before the age of 3.5 years, with 75% of children in the age group 2.5–3.5 years found to be seropositive [34]. In another report, the seroprevalence of HCoV-NL63 in 6–12-month-old children was 28.6–40.0% [35]. Maternally acquired antibodies were present in newborns and usually decreased within the first 4–5 months of life [34,35]. Three studies have calculated the incidence of HCoV-NL63 infections [5,19,36]. The design of these studies varied, however, and differences in the calculated incidences were reported. The comprehensive study by van der Hoek and colleagues [5] calculated the overall annual incidence in outpatients as seven per 1000 children per year, with the hospitalization rate estimated at 22 per 100,000 children; in contrast, a hospitalization rate of 224 per 100,000 (children younger than 6 years old) as previously reported [19].

At present, the accuracy of HCoV detection is hampered by three difficulties. First, the suitability of the clinical samples examined: a recent study has demonstrated that there are differences between respiratory samples collected by nose/throat swabs, and those collected by nasopharyngeal aspiration, specifically regarding their usefulness in detecting and identifying respiratory pathogens [37]. The second problem is that diagnostic tests for HCoVs are not frequently used in the routine testing for respiratory viruses, which probably results in the percentage of HCoVs infections being greatly underestimated [38]. Lastly, over the years several molecular methods of variable sensitivity were used to determine the incidence of the virus [39–41]. Unless sensitive and specific nucleic acid amplification tests are used for the detection of CoVs (including those infections with low viral load) as part of a respiratory virus surveillance strategy, the CoVs will always be underdiagnosed [42].

Even though the majority of initial reports identified winter as the peak season for HCoV-NL63 infections, other studies reported seasonal variations for HCoV-NL63 infections, including spring and autumn for China [19], and summer for Taiwan [43]. Also, although a study from Thailand, with its tropical climate, showed year-to-year variation in prevalence of HCoV-NL63 infections, the virus was detected throughout the year [44]. A recent comprehensive 2-year population-based study, using data from different countries, on children under 3 years of age with lower respiratory tract infection (LRTI) shows that HCoV-NL63 infections peak in winter months. This study also shows large year-to-year differences in the frequency of HCoV-NL63 infections, with results indicating an interepidemic period of 2 years [5].

## Respiratory clinical findings

Scientific and clinical evidence show that HCoV-NL63 infects both the upper and lower respiratory tract [36], causing symptoms similar to those associated with HCoV-229E and HCoV-OC43. Commonly, patients diagnosed with HCoV-NL63 infections of the upper respiratory tract present with mild symptoms, such as fever, cough, sore throat and rhinitis [18]. It is important to remember though, that the reports of symptoms in young children, who represent the majority of cases, are based mainly on parental observations, which often do not include possible subjective signs and symptoms. In addition, most studies are case reports of patients who have been hospitalized for acute respiratory tract infections (and other milder symptoms are ignored in favor of treating the more severe ones) and studies are often limited by small sample sizes or short periods of assessment [36]. For these reasons the involvement of HCoV-NL63 in many other diseases could be overlooked.

Recent studies have reported an association between HCoV-NL63 infection and more severe LRTIs [21,30,45]. LRTIs are the leading cause of morbidity in children younger than 5 years of age worldwide [46,47]. A recent study by Dominguez and colleagues reports that even though HCoV-NL63 and HCoV-OC43 were equally prevalent during their 1-year study period, HCoV-NL63 was more often associated with more severe LRTIs and subsequent hospitalization [21]. One of the most worrying clinical diagnoses of HCoV-NL63 infection is bronchiolitis, an inflammation of the membranes lining the bronchioles [22,24,25,30,45,48,49], and although a study of children hospitalized with fever and acute respiratory symptoms in China did not report an association of HCoV-NL63 with bronchiolitis [19], it still believed to be one of the presenting symptoms.

Several research groups have linked HCoV-NL63 infections to croup [5,17,19,28,30,43,50–52]. Croup children present with pharangitis, sore throat and hoarseness of voice, and infected children are considered for hospitalization. The chance of developing croup is 6.6-times higher in HCoV-NL63-infected children than in HCoV-NL63-negative children [30]. Another study reports that HCoV-NL63, when compared with other respiratory viruses, is the virus second-most commonly associated with young children (median age 13 months) hospitalized with croup [17]. In fact, HCoV-NL63 appears to be more frequently associated with croup than HCoV-229E and HCoV-OC43 [7]. Previously, the etiological agent of croup was generally assumed to be one of the more well-known respiratory viruses, such as the parainfluenza viruses, but it is now clear that HCoV-NL63 also plays a major role in this disease [9].

It is important to remember that since HCoVs (including HCoV-NL63) are also frequently detected in asymptomatic individuals [44,53,54], the studies that lack of specimens from healthy individuals limit the inference of a disease association. A recent study, however, reported that for all four circulating HCoVs combined, the detection frequency in samples from patients with URTIs and LRTIs exceeds the proportion seen with samples taken from patients with no respiratory symptoms; this provides epidemiological evidence for the role of HCoVs in the etiology of respiratory disease [55]. Furthermore, since it has previously been shown that HCoV can be detected in clinical samples 14 days after illness [56], the possibility that some of the control patients in the earlier studies may have been shedding HCoV following an earlier symptomatic infection cannot be excluded.

## Nonrespiratory clinical findings

To date, one group has reported an association between HCoV-NL63 and Kawasaki disease, a form of early childhood systemic vasculitis that presents as prolonged fever, polymorphic exanthem, oropharyngeal erythema and bilateral conjuctivitis [57]. In view of this paper, the editorial by McIntosh [8] made a compelling argument for a possible association between respiratory HCoVs and Kawasaki disease, motivating further study using broader epidemiological and nonepidemiological criteria. All subsequent reports, however, have conclusively demonstrated that no statistical significant link between HCoV-NL63 and Kawasaki disease exists [8,58–64].

HCoV-NL63 infections have previously been associated with gastrointestinal findings [21,25,29,65–67]. However, this is not unique to HCoV-NL63 infections, as coronavirus-like particles [21], as well as SARS-CoV and HCoV-HKU1 RNA [68,69] have been previously detected in patient diarrheic samples. These manifestations appear to be a direct consequence of viral invasion of the intestinal mucosa [7]. In one study, 4 out of 878 stool samples from children with acute gastroenteritis tested positive for HCoV-NL63, and these samples are usually positive for other gastroenteritis viruses as well [70]. Another study using stool samples collected from 479 patients, reported the absence of HCoV-NL63 [71]. However, this study had several shortcomings, including a short study period, the absence of a control group without gastrointestinal disease, and specimen selection that might be biased towards individuals with more severe disease. Also, the majority of patients included were adults (older than 18 years of age); this is a problem since it has previously been shown that young children are more susceptible to HCoV-NL63 infections. Therefore, the findings of this study cannot exclude the role of HCoV-NL63 in gastrointestinal disease [71]. Thus far, data show that HCoV-NL63 may, at most, have a minor etiological role in acute gastroenteritis in children, but since other viruses are frequently associated with gastroenteritis in HCoV-NL63 infected individuals, the exact role of HCoV-NL63 is not clear.

## Coinfections with other respiratory viruses

Coinfections of HCoV-NL63 and other respiratory viruses, including other HCoVs, influenza A virus, respiratory syncytial virus, parainfluenza virus and human metapneumovirus (hMPV), are common [19,23,27,30,31,43,44,72,73]. Interestingly, coinfected patients are more likely to be hospitalized, indicating the severity of this kind of superinfection [74]. In a study from Germany, respiratory syncytial virus A and HCoV-NL63 was the most common coinfection identified in children less than 3 years of age. This is probably due to the high incidence of respiratory syncytial virus A in winter and the overlap in the seasonality of the viruses [30]. Co-infection in hospitalized children with HCoV-NL63 and bocavirus is reported [75]. The viral load of HCoV-NL63 is lower in coinfected patients than in patients infected with HCoV-NL63 only [5,30]. The clinical significance of these coinfections are not clear, but various plausible explanations for the lower HCoV-NL63 viral load have previously been discussed: HCoV-NL63 causes the initial infection that weakens the immune system enough for a second viral infection to gain a foothold. By the time this second infection shows symptoms, the HCoV-NL63 infection might have already been brought under control by the host immune system; HCoV-NL63 and the other virus may be in competition for the same cellular receptor or target cell in the respiratory organs; the activation of the innate immune response triggered by the second respiratory virus may cause inhibition of HCoV-NL63; or prolonged persistence of HCoV-NL63 at low levels of expression [30,74]. Even though these reports may reflect biological complexity or interaction, it is important to keep in mind that virtually all the published studies comprise solely of cohorts of children hospitalized for ARTI and thus are extremely biased.

## Future perspective

New data concerning HCoV-NL63 and other HCoVs indicate that HCoVs may be more clinically important in children and the immunocompromised than previously thought. Since vaccines are not currently available for these respiratory viruses, it is necessary to monitor epidemic patterns and investigate the spread of respiratory infections to efficiently identify, control and prevent epidemics. More comprehensive population-based studies are required to determine the involvement of HCoV-NL63 in other body systems. Also, the development of technologies to accurately identify HCoV-NL63 infections will shed light on the true incidence of this virus in the human population.

Finally, a detailed manipulation of the HCoV-NL63 genome to understand the role of the HCoV-NL63 viral genes in pathogenesis and replication, and for the subsequent development of HCoV-NL63 as a vaccine vector, is needed. This, however, is hampered by the poor growth of the virus in cell culture, as well as the lack of an appropriate animal model. The recent development of the first full-length infectious clone of HCoV-NL63 allows for the systematic experimental study – genes can be modified and/or deleted from the genome – of the functions of the various corresponding HCoV-NL63 proteins, which will lead to a better understanding of the role of the viral genes in infectivity and pathogenicity. This manipulation of the virus genome, in turn, provides a reverse genetics platform that can lead to the development of HCoV-NL63-based vector vaccines [76].

## Conclusion

The detection of HCoV-NL63 in samples collected in 1981 [36] and 1988 [16] shows that the virus has been circulating and causing disease in the human population for a long time. However, the discovery of HCoV-NL63 and other novel HCoVs does not necessarily represent a sudden increase in emerging infections by ‘new’ CoVs. Of the CoVs isolated from patients in the 1960s, at least four were shown to be serologically distinct [77–79]. Unfortunately, these clinical samples were lost before they could be characterized and it will never be known whether these ‘old’ HCoVs and the current ‘new’ HCoVs represent the same strains [80]. HCoV-NL63 infections vary in frequency between years, but appear to peak during the winter months. HCoV-NL63 causes LRTIs and URTIs in 1.0–9.3% of children, the elderly and the immunocompromised, with symptoms ranging from mild to severe. Although unlikely, the high prevalence of coinfections of HCoV-NL63 and other respiratory viruses increases the chances of genetic recombination between these viruses in the host. In theory, these types of recombination events could enable highly pathogenic virus variants to arise [81]. Current data clearly show that HCoV-NL63 is clinically more important that previously suspected.

**Table 1.** Incidence of HCoV-NL63 in clinical samples.

**Country**	**Method used for detection**	**Incidence (%; sample size)**	**Ref.**
Australia	RT-PCR	3.30 (234)	[31]

Belgium	RT-PCR	2.30 (309)	[29]

Canada	RT-PCR	3.60 (525)	[18]

France	RT-PCR	9.30 (300)	[25]

Germany	Real-time RT-PCR	5.20 (949)	[28,30]

China	RT-PCR	2.60 (587)	[19]

Italy	RT-PCR	1.86 (150)	[27]

Japan	RT-PCR	1.20 (419)	[24]

Japan	RT-PCR	2.50 (118)	[22]

South Korea	RT-PCR	1.60 (515)	[20]

Taiwan	Quantitative RT-PCR	1.30 (539)	[43]

Thailand	RT-PCR	0.42 (1890)	[44]

South Africa	RT-PCR	8.30 (238)	[26]

Switzerland	RT-PCR	7.00 (82)	[23]

Sweden	RT-PCR	6.00 (212)	[32]

USA	RT-PCR	2.20 (1683)	[21]

South Korea	Multiplex RT-PCR	16.5 (182)	[17]

Germany	Real-time RT-PCR	3.90 (1756)	[5]

Norway	Multiplex real-time PCR	4.50 (536)	[33]

*RT-PCR: Reverse transcription PCR.* *Modified from* [74].

**Executive summary**

**Prevalence & seasonal incidence**

- Human coronavirus (HCoV)-NL63 has a worldwide distribution.
- HCoV-NL63 infections peak in winter and have an interepidemic period of 2 years.

**Respiratory clinical findings**

- HCoV-NL63 infects both the upper and lower respiratory tracts.
- Infection with HCoV-NL63 is often associated with more severe lower respiratory tract infections and subsequent hospitalization.
- Lower respiratory tract symptoms include croup and bronchiolitis.

**Nonrespiratory clinical findings**

- The majority of studies did not find an association between HCoV-NL63 infection and Kawasaki disease.
- HCoV-NL63 has been detected in a small number of diarrhea stool samples.

**Coinfections with other respiratory viruses**

- Coinfections with other well known respiratory viruses are very common.
- HCoV-NL63 viral load is higher in patients infected with HCoV-NL63 alone.
- Patients with coinfections are more likely to be hospitalized.

**Future perspective**

- More comprehensive population-based studies are needed to determine the involvement of HCoV-NL63 in the infection of systems other than the respiratory tract.
- The development of the HCoV-NL63 infectious clone will greatly aid in understanding this virus.
- An animal model is required for virus study and vaccine development.
